# Different types of cell death and their interactions in myocardial ischemia–reperfusion injury

**DOI:** 10.1038/s41420-025-02372-5

**Published:** 2025-03-05

**Authors:** Bingxin Du, Qiang Fu, Qin Yang, Yeying Yang, Rui Li, Xu Yang, Qingrong Yang, Shuo Li, Jinwei Tian, Huibin Liu

**Affiliations:** 1 Department of Pharmacy, The Second Affiliated Hospital of Harbin Medical University, Harbin, China; 2 Department of Chinese Formulae, Heilongjiang University of Chinese Medicine, Harbin, China; 3 Heilongjiang Provincial Key Laboratory of Panvascular Disease, Harbin, China; 4 Department of Cardiology, The Second Affiliated Hospital of Harbin Medical University, Harbin, China

**Keywords:** Heart failure, Cell death

## Abstract

Myocardial ischemia–reperfusion (I/R) injury is a multifaceted process observed in patients with coronary artery disease when blood flow is restored to the heart tissue following ischemia-induced damage. Cardiomyocyte cell death, particularly through apoptosis, necroptosis, autophagy, pyroptosis, and ferroptosis, is pivotal in myocardial I/R injury. Preventing cell death during the process of I/R is vital for improving ischemic cardiomyopathy. These multiple forms of cell death can occur simultaneously, interact with each other, and contribute to the complexity of myocardial I/R injury. In this review, we aim to provide a comprehensive summary of the key molecular mechanisms and regulatory patterns involved in these five types of cell death in myocardial I/R injury. We will also discuss the crosstalk and intricate interactions among these mechanisms, highlighting the interplay between different types of cell death. Furthermore, we will explore specific molecules or targets that participate in different cell death pathways and elucidate their mechanisms of action. It is important to note that manipulating the molecules or targets involved in distinct cell death processes may have a significant impact on reducing myocardial I/R injury. By enhancing researchers’ understanding of the mechanisms and interactions among different types of cell death in myocardial I/R injury, this review aims to pave the way for the development of novel interventions for cardio-protection in patients affected by myocardial I/R injury.

## Fact

- Myocardial reperfusion is the primary treatment for myocardial ischemia.
- Multiple modes of cell death, such as apoptosis, necroptosis, autophagy, pyroptosis, and ferroptosis, occur following myocardial reperfusion.
- The intricate connections among these pathways reveal cross-regulation.

## Open questions

- Exploring the mechanisms of cell death in myocardial I/R injury in order to uncover novel cardioprotective interventions.
- The complex collaboration of different cell death pathways in myocardial I/R injury requires a clearer explanation.
- Further development and evaluation are needed to determine the potential of specific inhibitors or activators targeting cell death pathways for clinical application.

## Introduction

Myocardial ischemia–reperfusion (I/R) injury is a critical condition that occurs when blood flow to the heart tissue is temporarily interrupted and subsequently restored [1]. This phenomenon is commonly observed in patients with coronary artery disease and can lead to severe damage to the myocardium. The restoration of blood flow during reperfusion, although essential for tissue survival, paradoxically exacerbates the injury and leads to cell death, resulting in adverse clinical outcomes [2]. Cardiomyocyte death constitutes a fundamental aspect of myocardial I/R injury. Comprehending the involvement and regulatory mechanisms of various types of cell death in myocardial I/R injury is of utmost importance for the development of effective interventions aimed at protecting the heart.

After reperfusion, elevated oxidative stress and mitochondria damage lead to increased cell death across various mechanisms. These mechanisms include necroptosis caused by heightened oxidative stress, protective autophagy triggered by mitochondria damage, excessive damaging autophagy exacerbated by further injury, pyroptosis initiated by inflammatory cascades and aggravated cell damage, and oxidative ferroptosis induced by increased oxidative stress and iron ion accumulation. Traditionally, these forms of cell death were considered distinct and regulated by separate signaling pathways [3]. However, emerging evidence now indicates a significant crosstalk and intricate interplay among these cell death mechanisms, blurring their traditional boundaries [4]. For instance, necroptosis can activate the occurrence of pyroptosis [5] and intracellular signal changes related to apoptosis may also affect the activation of pyroptosis [6]. Autophagy can inhibit apoptosis by eliminating damaged mitochondria, yet excessive autophagy may also induce apoptosis [7]. Furthermore, reactive oxygen species (ROS) generated during these forms of death can disrupt the intracellular oxidative balance, leading to ferroptosis [8]. These findings underscore the necessity for a comprehensive understanding of the interplay among different types of cell death in myocardial I/R injury, in order to develop targeted interventions that can effectively address this complex process.

In this study, we aim to provide a thorough exploration of the molecular mechanisms and regulatory patterns involved in these diverse forms of cell death in myocardial I/R injury. By elucidating the complex interactions and shared signaling pathways, we hope to shed light on the collective contribution of these forms of cell death to the pathogenesis of myocardial injury. This knowledge will not only enhance our understanding of myocardial I/R injury but also pave the way for the development of novel therapeutic strategies aimed at preserving cardiac function and preventing adverse outcomes in individuals with coronary artery disease.

## Pathophysiological mechanism of myocardial I/R injury

Myocardial I/R injury obtains complex pathogenesis, including oxidative stress, the opening of mitochondrial permeability transition pore (MPTP), and local inflammatory response [9]. As described, reperfusion serves as an effective mean to salvage the myocardium, yet it also amplifies oxidative stress, leading to cell injury and even death. Oxidative stress generates excess ROS which causes lipid peroxidation, and subsequently induces different types of cell death. Rapid restoration of pH, MPTP, mitochondrial plasma membrane depolarization, and oxidative phosphorylation during reperfusion result in ATP depletion and further cell death. In addition, I/R can lead to myocardial stunning, no-reflow phenomenon, reperfusion arrhythmia and potentially lethal reperfusion injury [10]. The loss of cardiomyocytes is a certainly outcome of reperfusion injury, which can occur through apoptosis, necroptosis, autophagy, and other pathways, contributing to irreversible damage and cardiac dysfunction. The table below (Table 1) outlines the roles of these various cell death forms in I/R and identifies the crucial factors that initiate and regulate each type of cell death.

**Table 1:** Cell death patterns in myocardial I/R injury.

Cell death	In myocardial I/R injury	Activating molecules	Key regulatory factors	Reference
Apoptosis	During the myocardial ischemia period, the process of apoptosis proceeds slowly. In the reperfusion phase, however, due to the exacerbation of oxidative stress, inflammatory responses, and mitochondrial damage, death receptors are activated, triggering both the extrinsic apoptotic pathway and the intrinsic apoptotic pathway, thereby accelerating the apoptotic process.	TNF-α, TNF-R, TRAIL, Fas, IRE1α, PERK, ATF4/6, Bcl-2, Bax, Bid, Bak, SIRT1	FADD, caspase-3/8, Apaf-1, cytochrome c, dATP/ATP, caspase-9, CHOP, NF-κB, IL-34, miR-484, MOMP, JNK	[14, 25–30, 34, 40, 43, 48, 50, 53]
Necroptosis	During the later reperfusion phase, necroptosis erupts. Massive amounts of inflammatory factors, like TNF-α, are generated, which activate RIPK1. When RIPK1 interacts with RIPK3 and MLKL, MLKL gets phosphorylated and then translocates to the cell membrane, thereby causing the cell membrane to be damaged. Meanwhile, the continuous opening of the mitochondrial MPTP releases CypD and other substances, which also facilitates the development of necroptosis.	TNF-α, PIPK1, PIPK3, MLKL, SIRT3, p53	TAK1, MPTP, CypD, Drp1, CaMKII, NF-κB, ARC, Parkin, caspase-3/8	[16, 59, 60, 62, 64, 65, 67, 71–75, 77]
Autophagy	During the ischemia period, autophagy gets activated as a result of the imbalance in intracellular energy metabolism and the build-up of metabolic waste. During the reperfusion stage, autophagy turns into a double-edged sword. While moderate autophagy remains protective, excessive autophagy can speed up cell death.	PI3K, AMPK, mTOR, HIF-1α, VDAC1, PERK, ATF6	ULK1, ATG13, FIP200, p62, Beclin1, complex I, IRE1α, ATF4, PINK1, Parkin, Bcl-2, BNIP3, AKT, OPA1, CHOP	[81, 82, 85, 87, 89, 91, 93, 96, 97, 102, 108, 109, 113, 116]
Pyroptosis	Pyroptosis initiates during the early stages of reperfusion, triggering acute I/R injury. It can be induced through two pathways: the caspase-1-dependent classical pathway and the caspase-4/5/11-dependent non-classical pathway.	LPS, NLRP3, Ca^2+^, ROS	IL-1β, IL-18, caspase-1, caspase-4/5/11,ASC, GSDMD, TLR4, MyD88, NF-κB, p65, p105, caspase-3, ZBP1, caspase-8	[19, 119–123, 125–127, 134, 135, 142, 143, 151, 154]
Ferroptosis	Ferroptosis is the predominant form of cell death in the late phase of myocardial I/R Injury. The mechanisms underlying ferroptosis involve complex interactions between iron accumulation, lipid peroxidation, and antioxidant system dysfunction.	Nrf2, Fe^3+^, PUFAs, NCOA4	ACSL4, System Xc-, GSH, GPX4, SLC7A11, SLC3A2, FTH1, transferrin receptor, six transmembrane epithelial antigen of the prostate-3, MLKL	[132, 161, 163, 165, 167, 168, 170, 173, 174, 181, 188, 194]

The role, activating molecules, and key regulatory factors of different cell death modes in myocardial I/R injury.

A single form of death has received significant attention, whereas, the form of death cannot be fully explored independently. The appreciation for the cross-regulation of cell death is steadily growing.

During I/R, different types of cell death occur in a specific sequence, accompanied by distinct dynamic changes. Initially, to cope with the hypoxia and energy metabolism disruptions during ischemia, autophagy is activated as a protective mechanism [11]. Autophagy helps maintain intracellular environmental stability by degrading damaged organelles and proteins through lysosomes. However, during the reperfusion phase, the role of autophagy shifts.

The restoration of blood flow introduces oxygen and nutrients back to the tissue but also generates ROS and other oxidative stressors [12]. These stressors activate autophagy-related protein (ATG) molecules, but excessive autophagy can lead to autophagic cell death, where cells essentially digest themselves [7]. As ischemia time prolongs and reperfusion begins, apoptotic pathways may be activated [13]. ROS production increases during reperfusion, and factors like intracellular calcium overload can activate both extrinsic and intrinsic apoptotic pathways [14]. Cleaved caspase-3 levels rise as reperfusion continues [15]. Under certain conditions, such as when the apoptotic pathway is inhibited or under intense inflammatory signals, necroptosis may occur [16]. Necroptosis shares some upstream signaling molecules with apoptosis but has a different execution stage, leading to cell membrane rupture, release of intracellular contents, and triggering an inflammatory response [17].

In the early stages of myocardial I/R injury, cells release inflammatory mediators, activating inflammasome-like NOD-like receptor family pyrin domain containing 3, which in turn activate caspase-1 and lead to pyroptosis [18]. Pyroptosis releases inflammatory factors like interleukin-1β (IL-1β) and interleukin-18 (IL-18), further exacerbating the inflammatory response [19]. Studies suggest that compared to apoptotic markers, pyroptosis may occur earlier and be more beneficial in the early stages of myocardial I/R injury [20].

Necroptosis is closely related to inflammation during myocardial I/R. Inflammation can directly trigger necroptosis, which typically occurs in the late phase of reperfusion [18]. Necroptosis increased significantly after oxygen-glucose deprivation and reoxygenation injury recovery. The phosphorylation of mixed lineage kinase domain-like protein (MLKL) also increased, indicating necroptosis [21].

During reperfusion, oxidative stress intensifies, intracellular iron metabolism is disrupted, and the antioxidant defense system, such as glutathione peroxidase 4 (GPX4), is damaged. This leads to the accumulation of lipid peroxidation products and the induction of ferroptosis [8]. Ferroptosis may emerge during prolonged reperfusion when intracellular redox state imbalance and lipid metabolism abnormality reach a critical level [22].

In brief, during I/R injury, these distinct types of cell death occur sequentially with unique dynamic characteristics, hinting at potential interactions among them. Recognizing these processes and their interplay is essential for the formulation of effective therapeutic approaches aimed at alleviating I/R injury.

## Apoptosis

### Mechanisms of apoptosis

Morphologically, apoptosis is characterized by DNA fragmentation, chromatin condensation, cytoplasmic compaction following plasma membrane blebbing, nuclear membrane rupture, elevated mitochondrial membrane permeability, and apoptosome formation [23]. It can be initiated through two well-defined pathways: extrinsic pathway mediated by death receptors and intrinsic pathway mediated by mitochondria. In addition, endoplasmic reticulum (ER) mediated apoptosis also plays an important role. Endoplasmic reticulum stress (ERS) occurs when the unfolded or misfolded proteins are accumulated in the ER [24].

Extrinsic pathway is triggered by transmembrane death receptors belonging to the tumor necrosis factor (TNF) receptor family (TNF-R, Fas, TNF-related apoptosis-inducing ligand (TRAIL)-receptor 1/2) [25]. Subsequently, it results in the recruitment of Fas-associated death domain protein (FADD) and activation of caspase-8 [14]. Caspase-8 then triggers caspase-3, resulting in apoptosis. In addition, the combination of TNF-α and TNF receptor induces two complexes, complex IIA and complex IIB, which also induce caspase-8/caspase-3-mediated apoptosis.

The formation of apoptosome (cytochrome c, apoptotic protease-activating factor-1 (Apaf-1), and dATP/ATP) is the key of the intrinsic pathway. Simultaneously, it will cause intracellular homeostatic imbalance caused by stimuli [26]. Apaf-1 plays a negative role in inhibiting myocardial I/R injury and attenuating apoptosis in ischemic cardiomyocytes through activating procasepase-9, triggering the cleavage and activation of caspase-9 and caspase-3 [27].

The function of ER fails to recover under prolonged or overwhelming ERS, resulting in apoptosis by influencing ER membrane resident proteins: inositol-requiring protein 1α (IRE1α), protein kinase RNA-like ER kinase (PERK), and activating transcription factor (ATF) 6 [28]. Long-term activation of the IRE1α is associated with the c-Jun N-terminal kinase (JNK) pathway to induce apoptosis [29]. PERK is a transmembrane protein of ER, which separates from 78 kDa glucose-regulated protein during prolonged ER stress and phosphorylates the eukaryotic translation initiator factor to activate the ATF4 and C/EBP homologous protein (CHOP) to induce apoptosis [30].

### Apoptosis in myocardial I/R injury

During the period of myocardial ischemia, the apoptotic process proceeds slowly. However, upon entering the reperfusion phase, oxidative stress, inflammatory responses, and mitochondrial damage intensify, leading to the activation of death receptors such as TNF-R and Fas. This activation triggers both the extrinsic (via caspase-8/3) and intrinsic (via cytochrome c) apoptotic pathways, thereby accelerating the apoptotic process [13, 14]. Apoptosis prevention has been shown to ameliorate I/R-related tissue damage [31].

Nuclear factor-κB (NF-κB) is activated and translocated to the nucleus, where it mediates the transcription of TNF-α and other proteins [32]. During myocardial I/R, activated NF-κB binds to the κB sites within the TNF-α gene promoter, enhancing its transcription. Elevated TNF-α levels further activate NF-κB in a positive feedback loop, augmenting the inflammatory response and triggering the extrinsic apoptotic pathway [33, 34]. In the pathological process of myocardial I/R injury, the nuclear translocation of NF-κB increases the release of pro-inflammatory cytokines, such as TNF-α and IL-6 [35, 36]. In the rat cardiomyocyte I/R injury model, the elevation of cytoplasmic NF-κB levels, coupled with a significant reduction in nuclear NF-κB levels achieved through the knockout of ubiquitin-specific protease 47, resulted in a decreased level of apoptosis in the cells subsequent to I/R injury [37]. Another study reveals that Rosmarinic acid can attenuate the infarct size and cardiomyocyte apoptosis in cardiac I/R injury by downregulating the NF-κB-mediated signaling pathway. This effect is achieved by inhibiting the levels of inflammatory cytokines, such as TNF-α and IL-6 [38]. Thus, the NF-κB/TNF-α pathway holds promise as an effective therapeutic target for myocardial I/R injury [39]. The TRAIL and FasL pathways induce the formation of a death-inducing signaling complex and activate caspase-8 through FADD, leading to apoptosis [40]. To date, TRAIL and Fas have been implicated in the pathogenesis of myocardial I/R [33, 41].

In the intrinsic apoptotic pathway, inhibiting cytochrome c has been shown to suppress cardiomyocytes apoptosis during myocardial I/R injury [42]. In addition, cytochrome c can also activate procasepase-9. The release of cytochrome c is regulated by pro-apoptotic proteins (e.g., Bax, Bak, Bid) and anti-apoptotic proteins (e.g., Bcl-2). Bcl-2 can inhibit the expression of Bax, thereby reducing the mitochondrial outer membrane permeability (MOMP). During myocardial I/R injury, the expression level of pro-apoptotic protein (Bax, caspase-3) increased significantly, while the expression of anti-apoptotic protein (Bcl-2) decreased [43]. In myocardial I/R injury, activated caspase-8 cleaves cytosolic Bid to produce truncated Bid, which translocates to the mitochondrial outer membrane, recruiting cytosolic Bax [44]. If a severe imbalance occurs between pro-apoptotic and anti-apoptotic proteins on the mitochondrial membrane, it can lead to mitochondrial depolarization and MOMP, ultimately activating the mitochondria-dependent apoptosis pathway [45]. Z-VAD.fmk, a pan-caspase inhibitor, binds to the catalytic site of caspase proteases and leads to a decrease in caspase-3 levels [46]. In a relevant study, it was demonstrated that in rats with myocardial I/R injury, Z-VAD.fmk could effectively inhibit caspase-3 and 8 activities, which subsequently led to a reduction in infarct size and an improvement in left ventricular function [47]. In addition, the suppression of caspase-3 and caspase-9 by miR-484 protects myocardial cells from I/R injury during cardiomyocyte apoptosis [48].

**Fig. 1:**
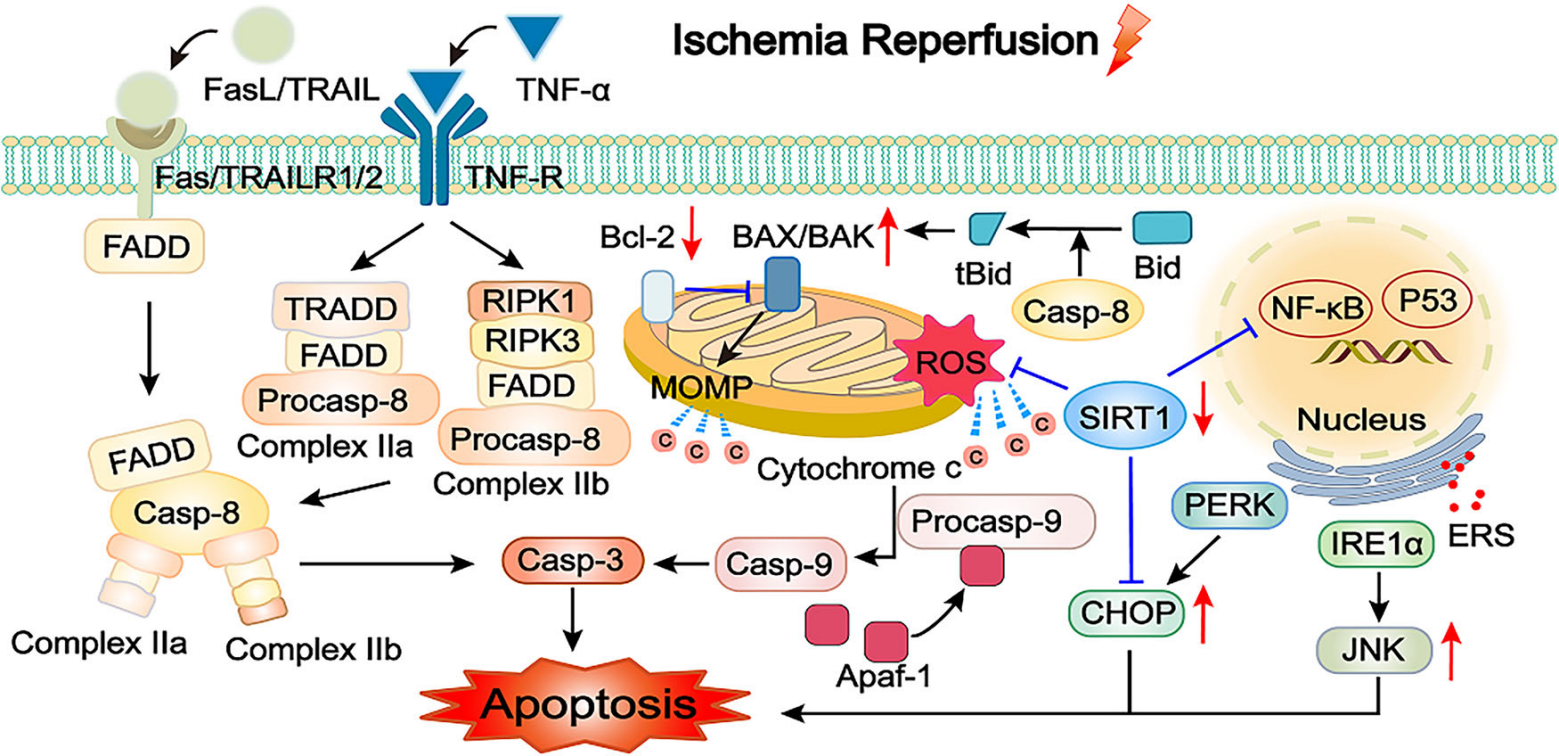
Mechanism of apoptosis in myocardial I/R injury. Apoptosis can be divided into an extrinsic pathway and an intrinsic pathway. TNF-α, FASL, and TRAIL interact with the related death domain to activate caspase-8 and caspase-3, inducing extrinsic apoptosis during myocardial ischemic injury. SIRT1 translocates to the nucleus to downregulate NF-κB, mediating TNF-α protein transcription. This process is impaired in myocardial I/R injury. SIRT1 downregulation is accompanied by a lower ratio of Bcl-2 to Bax and a higher MOMP following myocardial I/R injury. Activated caspase-8 cleavages cytosolic Bid to produce truncated Bid to increase BAX on mitochondria. Mitochondrial stress and MOMP can produce cytochrome c, then activate caspase-9 and caspase-3 in intrinsic apoptosis. Apaf-1 can promote the expression of procaspase-9 and facilitate the progression of apoptosis. Myocardial I/R injury can induce apoptosis by stimulating ERS, which induces the IRE1α/JNK and PERK/CHOP pathways. At the same time, SIRT1 can directly inhibit myocardial I/R injury-induced CHOP upregulation. FasL Fas ligand, TRAIL TNF-related apoptosis-inducing ligand, TNF-α tumor necrosis factor-α, FADD Fas-associated death domain protein, Casp caspase, TRADD TNF receptor-associated death domain, RIPK1/3 receptor-interacting protein kinase 1/3, SIRT1 silent information regulator transcript 1, NF-κB nuclear factor-κB, MOMP mitochondrial outer membrane permeabilization, Apaf-1 apoptotic protease-activating factor-1, tBid truncated Bid, ERS endoplasmic reticulum stress, IRE1α inositol-requiring protein 1α, PERK protein kinase RNA-like ER kinase, CHOP C/EBP homologous protein.

Post-I/R, ROS disrupts the balance between the oxidation and antioxidant systems, resulting in oxidative stress and cell damage. Preventing oxidative stress and ERS can protect against myocardial I/R injury [49]. JNK is activated in cardiac I/R injury. The inhibition of the IRE1α/JNK signaling axis exerts the function of cardio-protection [50]. Furthermore, PERK, as one of the ERS targets, is downregulated to alleviate ER stress-induced apoptosis in cardiomyocytes [51]. ERS-induced apoptosis is significantly increased following myocardial I/R injury. The expression of 78 kDa glucose-regulated protein, ATF6, CHOP in the myocardial I/R injury is significantly increased, which indicated that all three signal pathways of ERS-induced apoptosis are activated in the myocardial I/R injury [52]. The activation of silent information regulator transcript (SIRT) 1/CHOP signaling pathway reverses ER stress-dependent apoptosis of cardiomyocytes [53]. Meanwhile, SIRT1, a number of sirtuins family, regulates important transcription factors involved in myocardial I/R injury, such as p53 and forkhead box transcription factor class O [54, 55]. Other sirtuins also exhibit beneficial effects in myocardial I/R injury [56, 57] (Fig. 1).

## Necroptosis

### Mechanisms of necroptosis

Necroptosis is a form of regulated cellular necrosis. It is mainly characterized by organelle swelling, plasma membrane, and organelle rupture, culminating in the leakage of cellular contents [58]. Traditionally, the abatement of necroptosis is mainly downmediated by the TNF-α/receptor-interacting protein kinase (RIPK) 1/RIPK3/MLKL signaling pathway in myocardial I/R injury [59]. The RIPK1-RIPK3 complex is formed following inhibiting transforming growth factor-activated kinase 1 (TAK1) leading to cardiomyocyte necroptosis [60]. At the same time, the opening of MPTP can result in mitochondrial swelling and promote necroptosis.

### Necroptosis in myocardial I/R injury

During the later reperfusion phase, necroptosis occurs [61]. The process involves the production of massive amounts of inflammatory factors, such as TNF-α, which activate RIPK1. Upon interaction between RIPK3 and MLKL, MLKL gets phosphorylated and subsequently translocates to the cell membrane, leading to membrane damage [17]. In addition, the persisitent opening of the mitochondrial MPTP releases cyclophilin D (CypD) and other substances, which further promote the progression of necroptosis.

Traditionally, necroptosis is mainly mediated by the TNF-α/RIPK1/RIPK3/MLKL signaling pathway. A recent study showed that resveratrol can reduce necroptosis by inhibiting TNF-α/RIPK1/RIPK3/MLKL signaling pathway in the experiment of myocardial I/R [59]. A recent study also demonstrated that ginsenoside Rg2 enhances the interaction of TAK1 with RIPK1 by promoting the phosphorylation of TAK1, subsequently diminishing the formation of the RIPK1-RIPK3 complex [62]. Although necroptosis can be regulated through the RIPK1/RIPK3/MLKL pathway, this pathway exhibits non-conservative behavior in myocardial I/R injury. An experiment showed that the inhibition of only RIPK3 can attenuate cardiomyocyte necroptosis [63]. A study in 2018 showed that RIPK3 does not require RIPK1 and MLKL in myocardial I/R injury-induced necroptosis, but requires dynamin-related protein 1 (Drp1) [64], which is a protein related to mitochondrial division. Inhibiting Drp1 can protect myocardial cells against damage following reperfusion through the inhibition of necroptosis [65]. Another experiment also clearly showed that Drp1 has an increasing trend in myocardial I/R injury, and vitexin can reduce myocardial damage by affecting mitochondrial function through the reduction of Drp1 expression [66]. Similarly, afterward it was found that the RIPK3/calmodulin-dependent protein kinase II (CaMKII) signaling pathway also plays an important role in myocardial ischemia [67]. CaMKII activity is a central mechanism for mitochondrial calcium influx in myocardial cell death, and inhibiting mitochondrial-targeted CaMKII can prevent or reduce myocardial cell death and heart failure induced by various forms of pathophysiological stress [68]. CaMKII also plays a crucial role in myocardial I/R injury [69]. The most prevalent subtype of CaMKII in the heart is CaMKII-δ. A recent study has shown that inhibiting the splicing variant CaMKII-δ9 of CaMKII-δ can suppress the activation of inflammatory mediator NF-κB and reduce the inflammatory injury caused by myocardial I/R injury [70].

**Fig. 2:**
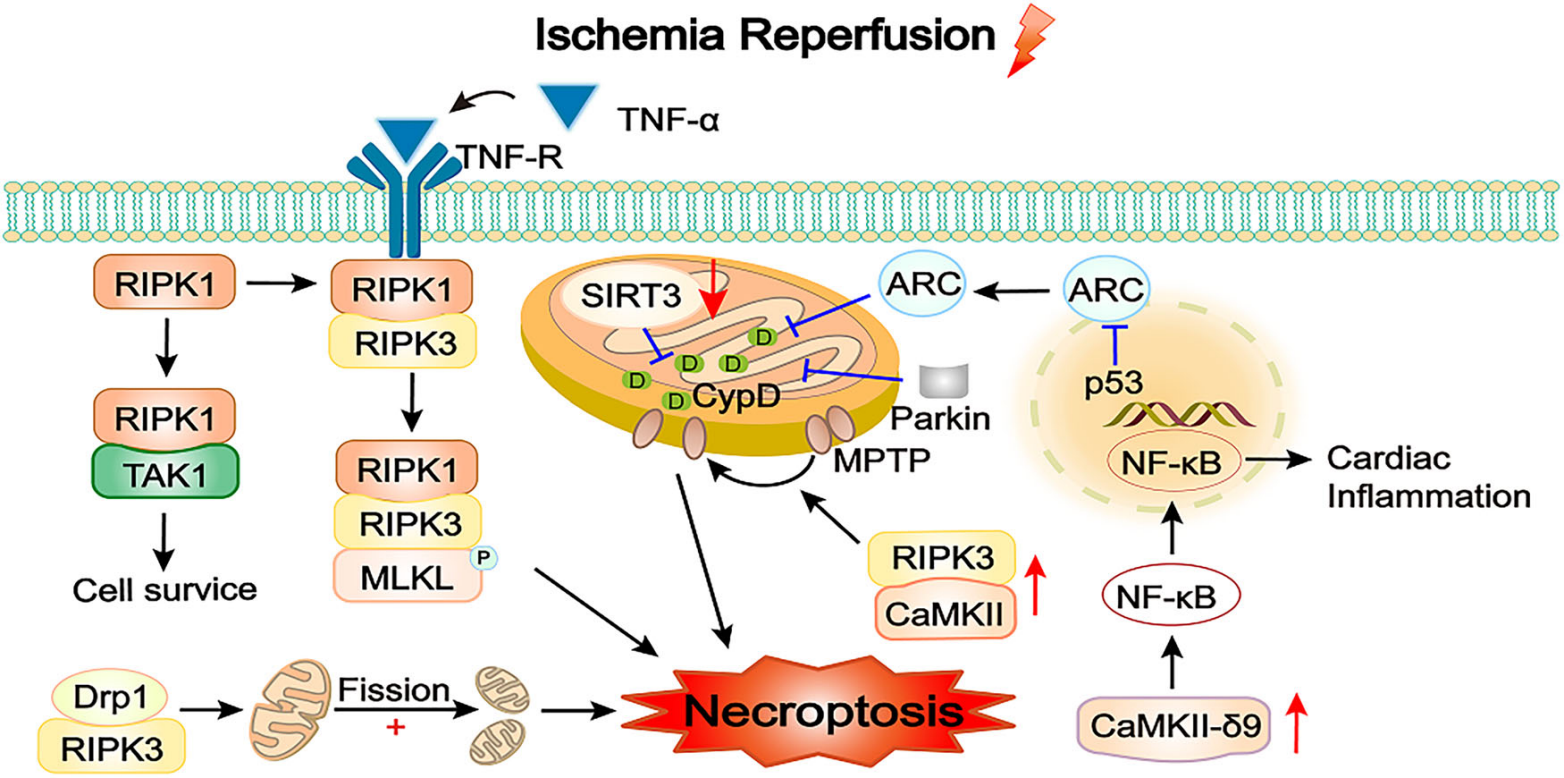
Mechanisms of necroptosis in myocardial I/R injury. Under normal conditions, TNF-α binding to its receptor promotes the combination of TAK1 and RIPK1. After TAK1 inhibition, RIPK1 dissociates from TAK1 and forms the RIPK1-RIPK3 complex. The TNF-α/RIPK1/RIPK3/MLKL pathway produces necrosomes and induces necroptosis. However, further studies have revealed that this process is not the only necroptosis pathway. RIPK3 can bind to Drp1, causing mitochondrial fission and affecting mitochondrial function. SIRT3, mainly localized to mitochondria, can act on CypD, inhibiting MPTP opening. Parkin inhibits MPTP opening by catalyzing the ubiquitination of CypD in the necrotic cascade, thereby inhibiting necrosis and improve cardiac function. ARC inhibits MPTP opening by inhibiting CypD. However, ARC expression is repressed by P53. CaMKII plays an important stimulative role in inhibiting MPTP opening pathway. CaMKII-δ9, the most widely distributed subtype of CaMKII, can induce the activation of the inflammatory mediator NF-κB and aggravate the inflammatory injury of myocardial I/R injury. TAK1 transforming growth factor-activated kinase 1, MLKL mixed lineage kinase domain-like protein, Drp1 dynamin-related protein 1, CypD cyclophilin D, CaMKII calmodulin-dependent protein kinase II, MPTP mitochondrial permeability transition pore, ARC apoptosis repressor with caspase recruitment domain.

Research has demonstrated that inhibiting the opening of MPTP during short-term ischemia has limited protective effects on myocardial cells after reperfusion, but it plays a more significant role in protecting against reperfusion injury after long-term ischemia [71]. This opening leads to irreversible depolarization of mitochondria, disrupts energy production, and triggers necroptosis. Simultaneously, the opening of MPTP in necroptosis is regulated by CypD, a factor associated with MPTP regulation. MPTP inhibitors primarily target CypD. Overexpression of SIRT3 prevents CypD acetylation, which limits the opening of MPTP and mitigates necroptosis [72]. The apoptosis repressor with caspase recruitment domain (ARC) prevents the opening of MPTP and reduce the rapid loss of ATP by targeting CypD, thereby improving myocardial injury and myocardial ischemia. ARC is a potential therapeutic target for protecting the heart during ischemia and oxidative stress-related heart disease [73]. It has been demonstrated that p53 inhibits ARC at the transcriptional level in myocardial I/R injury [74]. Parkin, an E3 ubiquitin protein ligase, catalyzes the ubiquitination of CypD and inhibits the opening of MPTP in the necrotic cascade [75]. In addition, the opening of MPTP can also be activated by CaMKII [76]. The correlation between mitochondrial function and necroptosis is highly significant. Therefore, exploring of mitochondrial-related proteins in-depth can identify more targets for the treatment of myocardial ischemia or cardiovascular diseases (Fig. 2).

### Interactions between necroptosis and apoptosis in myocardial I/R injury

Apoptosis and necroptosis are two significant forms of programmed cell death, with a closely intertwined and complex relationship. Caspase-8 plays a crucial regulatory role in this context. Upon receiving apoptotic signals, caspase-8, as a key protease initiating apoptosis, is activated [77]. The activation leads to the cleavage and activation of a series of downstream caspases family members, such as caspase-3. Simultaneously, activated caspase-8 also inhibits the formation of the necrosome, a key structure in necroptosis [16]. The formation of the necrosome, typically composed of RIPK1, RIPK3, and MLKL through their mutual interactions and aggregations [78], is a central step in the occurrence of necroptosis.

When caspase-8 is active, it cleaves key proteins such as RIPK1 and RIPK3, preventing their interactions and aggregations, thereby effectively inhibiting the formation of the necrosome and blocking the progression of necroptosis [79]. Conversely, when certain cellular factors downregulate caspase-8 activity, a significant shift in the cell death mode occurs. Since caspase-8 fails to effectively inhibit necroptosis, proteins like RIPK1 and RIPK3 can interact and aggregate to form the necrosome [77]. In this situation, due to the insufficient activity of caspase-8, the downstream apoptosis-related caspases cannot be fully activated, inhibiting the process of apoptosis and making the cell more susceptible to necroptosis. By targeting specific components of these pathways, such as RIPK1, RIPK3, or caspase-8, it may be possible to develop new therapeutic strategies to reduce myocardial damage and improve outcomes in patients with myocardial I/R injury.

## Autophagy

### Mechanisms of autophagy

Autophagy is a crucial metabolic process that involves breaking down aging or damaged proteins and organelles into amino acids and fatty acids for energy generation and recycling. It serves as a physiological mechanism for maintaining normal intracellular activity. Macroautophagy, the most significant type of autophagy, transports cytoplasmic components to lysosomes via autophagosomes, which then fuse with lysosomes [80].

Autophagy is regulated by ATG manipulation, with the ATG1 homolog UNC-51-like kinase (ULK) complex serving as the starting material for autophagosome formation. ULK complex consists of several individual components, including ATG13, family interacting protein of 200-kDa (FIP200), and others like ULK1/2 and ATG101, each playing specific functions and regulatory roles [81]. FIP200, serving as a scaffold protein, is required for the assembly and stabilization of the ULK complex to ensure the proper functioning of ULK1/2 and ATG13 in autophagosome formation [82]. As an essential part of the ULK complex, ATG13 plays a crucial role in regulating autophagosome formation by enhancing the kinase activity of ULK1/2 and interacting with other ATG proteins [83]. Experimental evidence, both from cellular studies and in vitro reconstituted reaction, has demonstrated that both FIP200 and ATG13 can independently augment the kinase activity of ULK1. However, the maximal stimulation of ULK1 kinase activity requires the presence of both FIP200 and ATG13 [84].

ULK1 is regulated by various signal kinases, including AMP-activated protein kinase (AMPK) and phosphatidylinositol-3-kinase (PI3K), which act as an upstream inhibitor and activator of mammalian target of rapamycin (mTOR). Essentially, mTOR inhibits autophagy activation by suppressing the formation of autophagy complex of FIP200-ULK1-ATG13 [85]. The phosphorylation state of ATG13 is closely regulated by mTOR complex 1. Under nutrient-rich conditions, mTOR complex 1 phosphorylates ATG13, which weakens its interaction with ULK1/2 and disrupts the formation of the ULK complex [86]. This phosphorylation event effectively inhibits the initiation of autophagy. Conversely, during conditions of nutrient deprivation or treatment with rapamycin, mTOR complex 1 activity is suppressed. This leads to a reduction in ATG13 phosphorylation, which in turn enhances its affinity for ULK1/2, triggering the initiation of autophagy [86].

In addition to regulating the signaling pathway of autophagy, the formation process of autophagy itself also affects its expression level. Beclin1 participates in the formation of Complex I (vacuolar protein sorting-associated protein 15-vacuolar protein sorting-associated protein 34-ATG14-Beclin1) [87]. Complex I plays a critical role in the regulation of autophagosome biogenesis, which localizes to the pre-autophagosome structure [88]. Furthermore, three ER membrane resident proteins (IRE1α, PERK, ATF6) not only regulate the protein folding ability of ER, but also modulate the process of autophagy [43, 89, 90]. Additionally, mitophagy is an effective means to maintain mitochondrial quality control to eliminate damaged and dysfunctional mitochondria. PTEN-induced kinase 1 (PINK1)-Parkin pathway is the classical way of mitophagy [91].

### Autophagy in myocardial I/R injury

During the ischemia period, autophagy gets activated as a result of the imbalance in intracellular energy metabolism and the build-up of metabolic waste [7]. At this point, the activity of mTOR is suppressed, and the ULK1 complex starts the autophagy process, clearing damaged organelles and proteins. Simultaneously, signals associated with ERS are turned on. During the reperfusion stage, autophagy turns into a double-edged sword. While moderate autophagy remains protective, excessive autophagy can speed up cell death [7].

It has been suggested that targeting upregulated autophagy could be a potential therapeutic approach for myocardial I/R injury [92]. The AMPK signaling pathway has been identified as an important target in autophagy. Activation of the AMPK/mTOR pathway can effectively increase autophagy and protect myocardial cells following I/R [93]. For example, melatonin, through AMPK signaling pathway, can act on optic atrophy 1 to improve mitochondrial fusion/mitophagy and reduce myocardial I/R injury [94]. Liraglutide, for instance, acted on the AMPK/mTOR pathway to enhance autophagy, improve autophagy flow, and alleviate myocardial cell damage [95]. A recent study on myocardial I/R showed that hypoxia-inducible factor-1α (HIF-1α)/Bcl-2/adenovirus E1B 19-kDa interacting protein 3 (BNIP3) pathway promotes autophagy to alleviate myocardial I/R injury [96]. BNIP3 is located in mitochondria in cardiomyocytes, which can release autophagy proteins such as light chain 3 and Beclin1, thus promoting mitophagy [97]. In a myocardial I/R injury rat model, western blot and immunofluorescence analyses confirmed significant elevation of HIF-1α and BNIP3 expression and a notable increase in the LC3II/LC3I ratio, and qPCR further verified significant upregulation of HIF-1α, BNIP3, and LC3 mRNA, while activation of the HIF-1α/BNIP3 pathway mediates mitophagy regulation to effectively reduce apoptosis in rat H9C2 cardiomyocytes [98]. A study has shown that directly activated autophagic flux reduces I/R-induced ROS levels and increases mitochondrial homeostasis during myocardial I/R injury [99].

However, some argue that an abnormal increase in autophagy during reperfusion could worsen myocardial injury [100]. In addition, autophagy becomes a damaging mechanism when autophagy occurs excessively. Reducing autophagy levels can mitigate oxidative stress, safeguard cardiac function, and alleviate cardiac remodeling [101]. PI3K/protein kinase B (AKT) is an important negative feedback regulatory signal pathway in myocardial I/R injury [102]. At the same time, promoting PI3K/AKT/mTOR phosphorylation can also inhibit expression of pro-inflammatory-related genes after myocardial I/R injury [73]. Several studies have shown that activating PI3K/AKT pathway can suppress autophagy, thereby improving cardiac function in I/R rats, alleviating oxidative stress injury, and exerting cardioprotective effect following myocardial I/R injury [103].

**Fig. 3:**
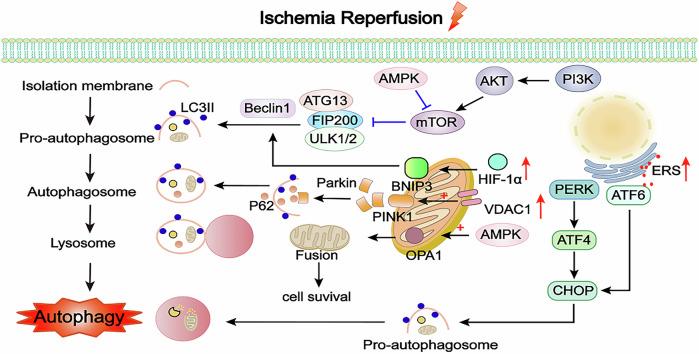
Mechanisms of autophagy in myocardial I/R injury. Autophagy is a physiological activity dependent on lysosomal degradation, but excessive autophagy can also lead to cell death. When the injury is mild, activating the autophagy pathway through activating the AMPK/mTOR signaling pathways can protect cardiomyocytes and alleviate myocardial I/R injury. AMPK also acts on OPA1 of mitochondria to improve mitochondrial fusion and alleviate myocardial I/R injury. BNIP3, localized only to mitochondria in cardiomyocytes, can activate the HIF-1α/BNIP3 pathway, increasing Beclin1 and promoting autophagy to protect cardiomyocytes. However, excessive autophagy can cause cardiomyocyte injury when the injury is aggravated. The PI3K/AKT/mTOR pathway is an important signal pathway for downregulating autophagy. The PINK1/Parkin pathway can activate cell autophagy and play an important protective role, but VDAC1 can strongly activate the PINK1/Parkin, inducing excessive autophagy, which can cause cell death. ERS can induce autophagy. PERK/ATF4/CHOP and ATF6/CHOP pathways are activated to induce autophagy after myocardial I/R injury. AMPK AMP-activated protein kinase, OPA1 optic atrophy 1, VDAC1 voltage-dependent anion channel 1, PINK1 PTEN-induced kinase 1, HIF-1α hypoxia-inducible factor-1α, BNIP3 Bcl-2/adenovirus E1B 19-kDa interacting protein 3, PI3K phosphatidylinositol-3-kinase, AKT protein kinase B, ATG13 autophagy-related protein 13, ULK1/2 UNC-51-like kinase 1/2, mTOR mammalian target of rapamycin, FIP200 family interacting protein of 200 kD, LC3II light chain 3 II, PERK protein kinase RNA-like ER kinase, ATF4/6 activating transcription factor 4/6.

Mitophagy is the selective autophagy of damaged mitochondria. Stimulated regulators of mitophagy may alleviate myocardial I/R injury by promoting mitochondrial quality control [104]. The main pathways implicated include those mediated by PINK1 and parkin proteins. PINK1 can improve mitochondrial dysfunction by alleviating cell hypoxia-reoxygenation (H/R) injury [105]. Later, several studies showed that PINK1/Parkin-mediated autophagy pathway played an important role in myocardial I/R injury [106, 107]. Voltage-dependent anion channel 1 (VDAC1) can strongly activate PINK1/Parkin pathway to induce autophagy, thus causing cell death. Furthermore, VDAC1 is a channel protein located in the outer membrane of mitochondria, which can regulate mitochondrial function [108]. When VDAC1 was knocked out, PINK1/Parkin pathway could be effectively inhibited. Therefore, it is considered to be a trigger of myocyte autophagy in the center of H/R injury [89]. ERS can induce autophagy. The interaction between the ER and mitochondria is closely linked, involving decreased expression of ATF6 and phosphorylation of PERK to lower CHOP expression, thus countering myocardial I/R injury by inhibiting ERS-induced autophagy [109]. PERK is an excellent and classical target related to ERS and autophagy. ER stress-activated autophagy through the PERK signaling. The PERK/ATF4/Beclin1 signaling is suppressed to inhibit ER stress-induced autophagy to alleviate myocardial injury [109] (Fig. 3).

### Interactions between autophagy and other forms of cell death in myocardial I/R injury

In myocardial I/R injury, autophagy interacts with other forms of cell death, such as apoptosis, necroptosis, and pyroptosis, in complex and often context-dependent manners. These interactions play a crucial role in determining the fate of cardiomyocytes and the overall outcome of myocardial I/R injury.

When the myocardial injury is mild, autophagy act as a protective mechanism by degrading non-functional cytoplasmic proteins and damaged organelles, such as mitochondria, which are major sources of ROS [99]. This process supplies essential nutrients for cell growth and survival and effectively inhibits apoptosis. By reducing ROS production, autophagy can prevent oxidative stress-induced apoptosis. Autophagy also directly modulates apoptotic signaling pathways by sequestering and degrading pro-apoptotic proteins, such as p53 and Bax, thereby inhibiting the activation of caspases and the execution of apoptosis [110]. Recent studies have shown that impaired autophagy induces cardiomyocytes apoptosis and necroptosis, and enhancing autophagic flux can inhibit them in myocardial I/R injury [111].

Autophagy dysfunction-mediated necroptosis mechanistically contributes to the loss of cardiomyocytes, adverse ventricular remodeling, and progressive heart failure following myocardial infarction [112]. Another study suggests that impaired autophagic flux during reperfusion resulted in p62 accumulation, while knockdown of p62 attenuated necroptosis during H/R injury [113]. It indicates that necroptosis and autophagy can regulate each other through mutual interference in myocardial I/R injury. Mechanistically, activation of necroptosis suppresses the fusion of autophagosomes with lysosomes [114]. Moreover, a study has also demonstrated that in the context of myocardial I/R injury, HSP70 can mitigate necroptosis of cardiomyocytes by suppressing autophagy during the myocardial I/R process [115].

However, during severe I/R injury, autophagy can switch from a protective to a pro-apoptotic role. Beclin1, a key component of the PI3K complex I, plays an important role in myocardial I/R injury. Normally, Beclin1 is involved in the formation of autophagosome. However, in the reperfusion period, its mutual inhibitory interaction with Bcl-2 reduces the function of Bcl-2, which promotes the formation of apoptosome and thereby makes autophagy change from a protective role to a pro-apoptotic one [116]. Downregulating Beclin1 can protects myocardium against myocardial I/R injury through inhibiting autophagy [117]. There is literature indicating downregulating Beclin1 and upregulating Bcl-2 expression to inhibit excessive autophagy and reduce apoptosis [118].

The roles of autophagy and pyroptosis are described in detail below.

## Pyroptosis

### Mechanisms of pyroptosis

Pyroptosis is an inflammatory programmed cell death characterized by chromatin concentration, DNA breakage, cell swelling, osmotic dissolution, plasma membrane rupture, and the release of pro-inflammatory cytokines such as IL-1β and IL-18 [19]. It can be induced through two pathways: the caspase-1-dependent classical pathway and the caspase-4/5/11-dependent non-classical pathway [119].

The caspase-1-dependent pathway typically involves the activation of caspase-1 through the formation of inflammasomes, such as the NLRP3 inflammasome [120]. Inflammasomes are multi-protein complexes that assemble in response to pathogen-associated molecular patterns or danger-associated molecular patterns. The NLRP3 inflammasome, composed of NLRP3, apoptosis-associated speck-like protein containing CARD (ASC), and procaspase-1, is particularly well-studied in innate immunity [121]. When activated, caspase-1 cleaves the gasdermin D (GSDMD) protein, causing pore formation in the cell membrane. This leads to cell swelling and lysis, as well as the processing and release of pro-inflammatory cytokines [122].

The activation of caspase-1 and the NLRP3 inflammasome can be initiated by various stimuli, including lipopolysaccharide (LPS) binding to toll-like receptor 4 (TLR4) on the cell surface. Mechanically, LPS binds to TLR4 on the cell surface, initiating the activation of myeloid differentiation primary response 88 (MyD88), a critical adapter protein in the TLR signaling cascade. MyD88 then interacts with interleukin-1 receptor-associated kinase and TNF receptor-associated factor 6, leading to the activation of NF-κB [123]. This, in turn, upregulates various pro-inflammatory cytokines and activates the NLRP3 inflammasome [124]. Therefore, the LPS-TLR4/MyD88/NF-κB/NLRP3 pathway initiates a cascade of molecular interactions that ultimately triggers pyroptosis through the activation of caspase-1 and the formation of pores in the cell membrane.

The non-classical pathway of pyroptosis involves direct recognition of cytoplasmic LPS by caspase-4/5/11, which leads to oligomerization and activation of these caspases. This, in turn, results in GSDMD cleavage and pore formation in the cell membrane [124], similar to the classical pathway. However, in this case, the activation of caspase-4/5/11 does not require the formation of inflammasomes or upstream signaling cascade involving TLR4, MyD88, and NF-κB [125]. Both the two main pathways ultimately result in GSDMD cleavage, pore formation in the cell membrane, and the release of inflammatory cytokines.

### Pyroptosis in myocardial I/R injury

Pyroptosis initiates during the early stages of reperfusion, triggering acute I/R injury. Both during the myocardial ischemia phase and reperfusion phase, the inflammatory response leads to an elevation of intracellular ROS levels [126, 127]. This increase, in turn, activates the NLRP3 inflammasome, which subsequently triggers the activation of caspase-1. The activation of the NLRP3 inflammasome-mediated caspase-1 signaling pathway plays a pivotal role in pyroptosis, exacerbating I/R injury [128]. Mitochondrial dysfunction further contributes to the release of ROS, activating the NLRP3 pathway and leading to pyroptosis in myocardial I/R injury [129]. Antioxidants, such as ethyl pyruvate, can mitigate this damage by scavenging ROS and reducing NLRP3 inflammasome-mediated pyroptosis [130]. Uric acid has been identified as another factor that aggravates myocardial I/R injury through the ROS/NLRP3 pathway [127]. Conversely, periostin has been shown to activate NLRP3, promoting caspase-1-mediated pyroptosis in myocardial I/R injury [131]. However, inhibiting NLRP3, a crucial protein in pyroptosis, can alleviate this process [132]. Hydroxysafflor Yellow A, for example, inhibits NLRP3 inflammasome activation, providing a protective effect against myocardial I/R injury [133]. The inositol 1,4,5-triphosphate receptor, an intracellular ion channel, plays a role in calcium homeostasis by releasing Ca^2+^ from the ER into the cytoplasm [134]. Inhibiting this receptor can reduce calcium overload and NLRP3/caspase-1 pathway activation in myocardial I/R injury [134]. In addition, the TLR4/MyD88/NF-κB/NLRP3 pathway is involved in myocardial I/R injury. Drugs like trimetazidine can alleviate myocardial I/R injury by preventing the upregulation of TLR4, MyD88, phospho-NF-κB p65, and the NLRP3 inflammasome [135]. Certain microRNAs, such as miR-148a carried by M2 macrophage-derived exosomes, can also mitigate myocardial I/R injury by downregulating thioredoxin-interacting protein and inactivating the TLR4/NF-κB/NLRP3 inflammasome signaling pathway [136].

The non-classical pyroptosis pathway, which is distinct from the classical pathway involving caspase-1, has garnered less attention in the context of myocardial I/R injury. However, recent research has shed light on its potential role in mitigating myocardial damage by demonstrating that reducing caspase-11-dependent pyroptosis can be beneficial in myocardial I/R injury [137]. In addition, the findings from other I/R injury models, such as retina, provide a compelling rationale to further investigate this pathway in the heart [138].

**Fig. 4:**
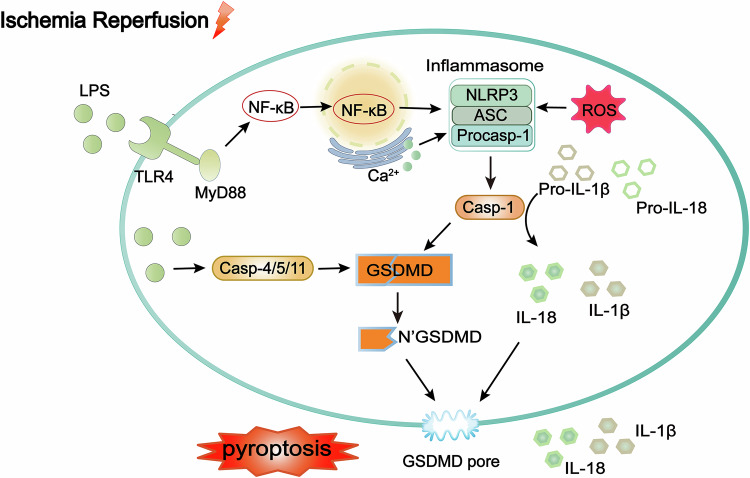
Mechanisms of pyroptosis in myocardial I/R injury. The caspase-1-dependent pathway involves the activation of caspase-1 through the formation of inflammasomes, such as the NLRP3 inflammasome. After injury, the inflammasome formed by NLRP3, ASC, and procaspase-1 is activated. Procaspase-1 is activated as caspase-1 to cleave GSDMD, causing pore formation in the cell membrane, leading to cell swelling and lysis. The GSDMD executes the inflammasome, inducing the release of inflammatory markers associated with inflammasome activation, such as IL-1β and IL-18, ultimately leading to pyroptosis. NLRP3 is activated by ROS burst from myocardial I/R injury and Ca^2+^ overload. Extracellular LPS activates pyroptosis via the TLR4/MyD88/NF-κB/NLRP3 inflammasome pathway, aggravating myocardial I/R injury. The non-classical pathway involves direct recognition of cytoplasmic LPS by caspase-4/5/11, resulting in oligomerization and activation, leading to GSDMD cleavage and pore formation in the cell membrane. LPS lipopolysaccharide, TLR4 toll-like receptor 4, MyD88 myeloid differentiation primary response 88, GSDMD gasdermin D, NLRP3 nucleotide-binding oligomerization domain-like receptor protein 3, ASC apoptosis-associated speck-like protein containing CARD, Casp caspase, NF-κB nuclear factor-Κb, IL-1β interleukin-1 beta, IL-18 interleukin-18.

As the executor of pyroptosis, GSDMD plays a critical role in cardiomyocyte pyroptosis under oxidative stress conditions [139]. Elevated serum GSDMD levels may serve as a diagnostic marker for myocardial I/R injury [140]. Recently, a new drug targeting GSDMD has been identified for the treatment of myocardial I/R injury [141]. In summary, these findings offer new insights into the treatment of myocardial I/R injury, as illustrated in Fig. 4.

### Interactions between pyroptosis and other forms of cell death in myocardial I/R injury

Pyroptosis and other forms of cell death exhibit complex interactions in myocardial I/R injury. Both apoptosis and autophagy interact with pyroptosis. Recent reports have also revealed the role of PANoptosis in myocardial I/R injury, clarifying the interaction mechanisms among pyroptosis, apoptosis, and necroptosis during myocardial I/R injury.

Previously, the mechanisms of action of apoptosis and pyroptosis in myocardial I/R injury have been elucidated. However, pyroptosis and apoptosis exhibit complex and profound connections, and they interact with each other. IL-1β-treated cells showed transient phosphorylation of NF-κB, p65 and p105, suggesting the activation of NF-κB in cardiac fibroblasts [142]. It shows that IL-1β, which may be released by pyroptosis through activating the NLRP3 inflammasome, can activate the NF-κB signaling pathway, thereby influencing the apoptotic pathway of cells. Caspase-3, a key protease in programmed cell death, is of great significance in both apoptosis and pyroptosis. During apoptosis, caspase-3 can be activated through the intrinsic and extrinsic pathways. In the context of pyroptosis, caspase-3 also plays a crucial part in participating in the cleavage of inflammatory proteins during this process [143]. Doxorubicin-induced caspase-3 activation triggered gasdermin E-dependent pyroptosis [144]. However, it was indicated in another article that caspase-3 cleavage at the Asp26 site in mice may interfere with caspase-1-mediated sequential cleavage, thereby inhibiting the production of mature IL-1β [145]. The two different outcomes of the above-mentioned functions emphasize the dual role of caspase-3 in the switch between apoptosis and pyroptosis. Future research is required to further clarify the mechanism by which caspase-3 mediates the switch between these two cell death pathways.

The regulation of pyroptosis and autophagy is interlinked. For instance, when mitophagy is inhibited, damaged mitochondria are unable to clear and release ROS, which can directly activate the NLRP3 inflammasome [146]. However, the precise balance between these two processes determines the cellular fate. Mitophagy pathway plays a role in inhibiting caspase-1 and alleviating pyroptosis [146]. Conversely, excessive autophagy can lead to cell death through autophagic cell death. The inflammatory signaling cascades activated during pyroptosis can also influence autophagy. For example, the NLPR3 inflammasome restricts autophagy through activated caspase-1 in prion peptide-infected microglia [147]. Activation of autophagy also leads to the degradation of the components of NLRP3, reducing the release of IL-1β and IL-18 in myocardial I/R injury [148]. In other words, inhibition of autophagy-dependent pyroptosis can exert myocardial protective effect including alleviating myocardial I/R injury and reducing infarct size in mice [149].

The concept of PANoptosis was officially proposed in 2019, due to the crosstalk of pyroptosis, apoptosis, and necroptosis [150]. PANoptosis has the characteristics of apoptosis, necroptosis, and pyroptosis. Subsequently, PANoptosome was identified as the interaction of RIPK1/3, caspase-8, NLRP3, ASC, and FADD [151]. PANoptosome was a momentous regulator in immune and inflammatory responses and a scoring method of immune cells in pan-cancer [152]. TAK1-deficient macrophages drive activation of PANopotsome [153]. The study explains that TAK1 is a switch of PANoptosis. Another switch is Z-DNA binding protein 1 (ZBP1), which could induce macrophage necroptosis [154]. Influenza A virus-induced PANoptosis through the ZBP1-PANoptosome, which is independent of MLKL [155]. However, unlike in macrophages, ZBP1 attenuates inflammation in cardiomyocytes by suppressing the RIPK3/NF-κB pathway [156]. The recent discovery of PANoptosis in cerebral I/R injury suggests that PANoptosis may exist in I/R models [157]. The latest results show that penehyclidine hydrochloride can reduce PANoptosis in myocardial I/R injury by decreasing ZBP1 [158]. Piezo1, as a novel cardiac mechanosensor, potentially promotes cardiac I/R injury. This might be achieved through the activation of cardiomyocyte PANoptosis mediated by caspase-8 [159].

## Ferroptosis

### Mechanisms of ferroptosis

Ferroptosis is an emerging concept in cell death biology, characterized as an iron-dependent, programmed form of cell death distinct from classical apoptosis and autophagy. Ferroptosis was first described in 2012 as a regulated form of cell death driven by iron-dependent lipid peroxidation [160]. Lipid peroxidation refers to the oxidative degradation of lipids, particularly polyunsaturated fatty acids (PUFAs), in cellular membranes [161]. This process results in membrane damage and ultimately cell death [161]. Ferroptosis is characterized by shrinkage of mitochondria, increased membrane density, and the preservation of nuclear integrity [160]. The mechanisms underlying ferroptosis involve complex interactions between iron accumulation, lipid peroxidation, and antioxidant system dysfunction [162].

Excess iron, whether from dietary intake, genetic mutations, or other sources, can accumulate in cells and tissues. Normally, transferrin binds to transferrin receptor on cell membrane and is internalized into endosomes. Within endosomes, Fe^3+^ is converted to Fe^2+^ by the six transmembrane epithelial antigen of the prostate-3 [163]. Ferritinophagy is a selective autophagy process. That is, cells recognize and enwrap ferritin through the autophagy mechanism to form autophagosomes [164]. After the autophagosomes fuse with lysosomes, the ferritin is degraded under the action of lysosomal hydrolases, and iron ions are released. The related mechanism mainly involves the mediation of nuclear receptor coactivator 4 (NCOA4). The C-terminal domain of NCOA4 can interact with the ferritin heavy chain 1 to transport ferritin to autophagosomes for degradation [165].

Free iron ions can directly oxidize lipids, particularly PUFAs, leading to the formation of lipid peroxides [166]. Lipid peroxides are unstable and can decompose to form additional ROS, further exacerbating oxidative stress. This iron overload can catalyze the Fenton reaction, which involves the conversion of hydrogen peroxide (H_2_O_2_) into highly reactive hydroxyl radicals (OH•). Hydroxyl radicals are powerful oxidizing agents that can damage lipids, proteins, and DNA, leading to cellular dysfunction and promoting the initiation and progression of ferroptosis. PUFAs could react with iron, leading to peroxidation of iron-dependent PUFA causes ferroptosis [166]. Acyl-CoA synthetase long-chain family member 4 (ACSL4) promotes PUFAs converted to PUFA-CoAs. Further, PUFA-CoAs are esterified to L-PUFAs, which are oxidized by lipoxygenases leading to the generation of lipid peroxidation [167]. Nuclear factor erythroid 2-related factor 2 (Nrf2) is regarded as a predominant regulator in the antioxidant response, since a multitude of its downstream target genes participate in averting or rectifying redox imbalances within the cell [168]. An upregulation of Nrf2-regulated gene transcription to counteract enhanced ROS generation and sustained oxidative stress [169]. The Nrf2 target plays a critical role in mediating the enzymes associated with the synthesis and metabolism of glutathione (GSH) and in the process of iron/heme metabolism [170–172].

The imbalance of antioxidant functions within cells is a significant contributor to ferroptosis. There are three major antioxidant systems associated with ferroptosis in vivo: the cystine/glutamate antiporter system (system Xc-)/GSH/GPX4 system, the nicotinamide adenine dinucleotide phosphate/ferroptosis suppressor protein 1/coenzyme Q10 system, and the guanosine triphosphate cyclohydrolase 1/tetrahydrobiopterin/dihydrofolate reductase system [173]. The latter two systems are less well extensively studied, and current research on ferroptosis in MIRI primarily focuses on the first system. In the Xc-/GSH/GPX4 system, GPX4 acts to scavenge membrane lipid hydroperoxides. However, when GPX4 is inactivated or when GSH is depleted, peroxides accumulate within the cell, triggering ferroptosis. The heterodimeric system Xc-, consisting of SLC7A11 and SLC3A2 [174], serves as a glutamate cystine antiporter responsible for intracellularly GSH synthesis. As a key inhibitor of ferroptosis, the light chain subunit SLC7A11 facilitates the import of cystine from the extracellular environment into the cell and synthesizing GSH. Inhibition of SLC7A11 activity or expression can lead to a depletion of GSH and an accumulation of ROS, ultimately inducing ferroptosis [175]. In contrast, the heavy chain subunit SLC3A2 mainly serves as a chaperone protein of SLC7A11 to provide SLC7A11 recruitment to the plasma membrane [176]. By supporting the function of SLC7A11, SLC3A2 indirectly contributes to the inhibition of ferroptosis. Inhibition of this system leads to GSH depletion. Moreover, inhibiting system Xc- causes excessive intracellular accumulation of glutamate, which affects the mitochondrial tricarboxylic acid cycle to stimulate mitochondrial ROS generation [177].

### Ferroptosis in myocardial I/R injury

Ferroptosis is the predominant form of cell death in the prolonged reperfusion phase of myocardial I/R Injury [22]. During the reperfusion period, the burst of ROS leads to an imbalance in intracellular antioxidant systems, like the system Xc-/GSH/GPX4 system [8]. This further promotes lipid peroxidation, especially intensifying the peroxidation of PUFAs on the cell membrane [8]. As a result, more iron ions within the cell are involved in the Fenton reaction, and ferroptosis consequently continues to progress, worsening the myocardial injury.

Transferrin-mediated iron is required for ferroptosis in myocardial I/R injury. A recent study revealed the relationship between transferrin and myocardial I/R injury. Resveratrol can effectively reduce the content of Fe^2+^ in cells and tissues by downregulating the expression of transferrin receptor 1, reduce cellular oxidative stress and upregulate GPX4 [132]. Cells acquire resistance against ferroptosis by Nrf2 pathway, thus enhancing iron storage capacity (elevates iron storage protein ferritin) and mitigating the decrease in GSH level [178]. In myocardial I/R injury, ferritinophagy also plays an important role in the release of iron ions. This part is detailed in “Interactions between ferroptosis and other forms of cell death in myocardial I/R injury”.

ACSL4, a key enzyme that regulates lipid composition, is activated to facilitate ferroptosis and myocardial I/R injury [179]. Inhibition of ACSL4 can effectively reduce myocardial I/R injury [180]. Imbalance of cellular antioxidant functions is a significant contributor to ferroptosis in myocardial I/R injury, mainly inhibited by the antioxidant system: system Xc-/GSH/GPX4 [181]. The heterodimeric system Xc-, consisting of SLC7A11 and SLC3A2, serves as a glutamate cystine antiporter responsible for intracellularly GSH synthesis. A study showed that overexpressed SLC7A11 selectively in cardiomyocytes could increase GSH levels and prevent cardiac ferroptosis [182]. Moreover, the low-level GSH in cell can inactivate GSH-dependent GPX4 to potentiating ferroptosis [183]. The SLC7A11/GSH/GPX4 pathway was repressed in myocardial I/R injury rat to induce ferroptosis, inducing myocardial injury [184]. GPX4 functions by scavenging membrane lipid hydroperoxides. However, when GPX4 is inactivated or it is depleted, peroxides are accumulated within the cell, triggering ferroptosis in myocardial I/R injury [185]. Naringenin can inhibit ferroptosis by regulating Nrf2/system Xc-/GPX4 axis, thus alleviating myocardial I/R injury [186]. Isoliquiritigenin has the ability to alleviate oxidative stress, mitochondrial damage and cardiomyocyte ferroptosis induced by I/R, thus reducing myocardial injury, and a key mechanism of which is triggering the Nrf2 pathway to prevent oxidative stress damage and cardiomyocyte ferroptosis resulting from I/R [187]. Moreover, the inhibition of Mucosa-associated lymphoid tissue lymphoma translocation gene 1 is capable of reducing myocardial ferroptosis induced by I/R via the enhancement of the Nrf2/SLC7A11 pathway [188]. A recently discovered antioxidant system has been found to play a role in ferroptosis. Ferroptosis suppressor protein 1 reduces coenzyme Q10 to dihydro-Q10, which can act as antioxidants to inhibit the accumulation of lipid peroxidation [157].

**Fig. 5:**
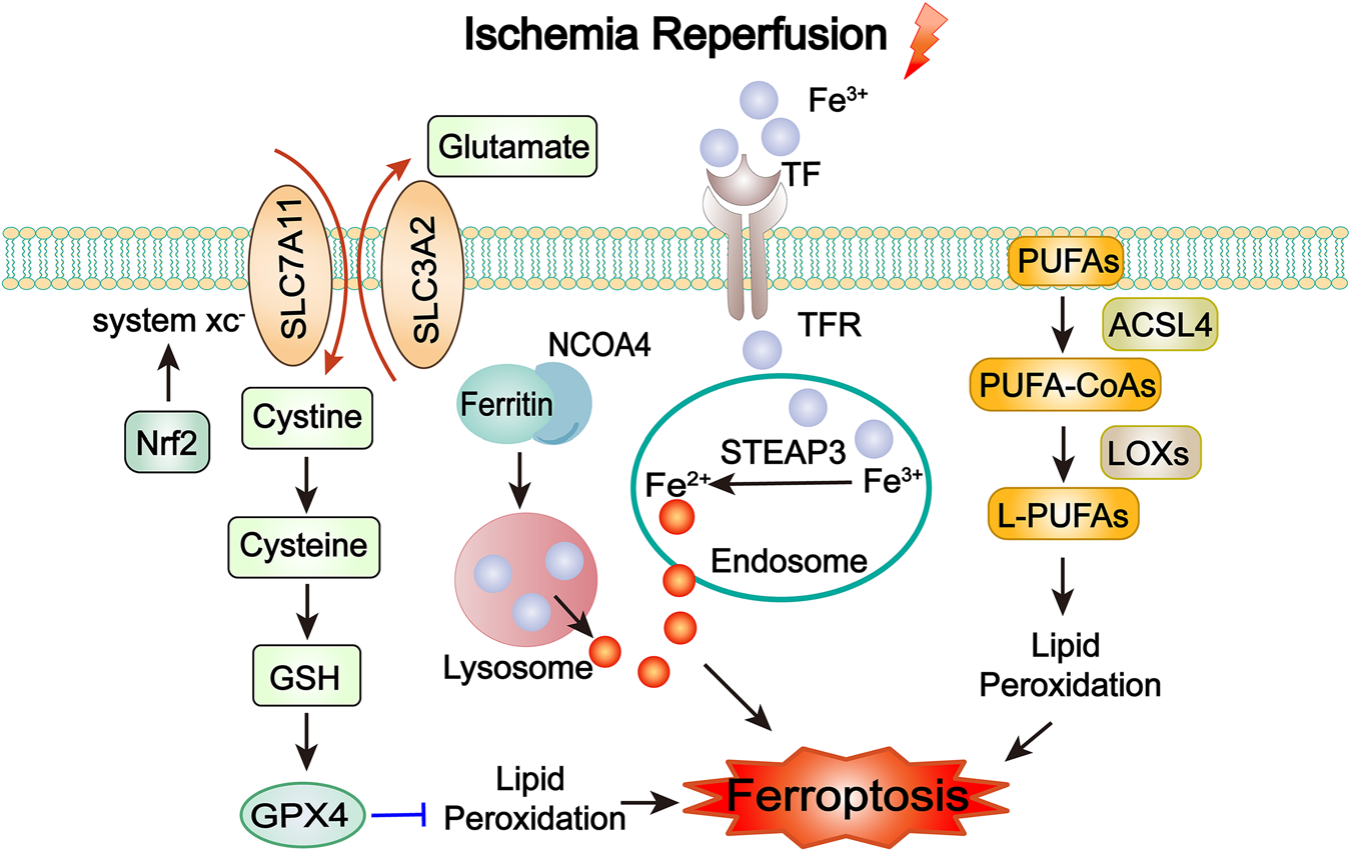
Mechanisms of ferroptosis in myocardial I/R injury. Ferroptosis is an oxidative death resulting from lipid peroxidation and iron overload. The system Xc-/GSH/GPX4 system is the main antioxidant system involved in the onset of myocardial I/R injury ferroptosis, inhibiting the increase in ROS and attenuating ferroptosis. Inhibition of this pathway can stimulate the onset of ferroptosis. Excessive labile iron ions generated after autophagy of ferritin can undergo a Fenton reaction with intracellular lipid peroxidation, leading to ferroptosis. PUFAs could react with iron, leading to peroxidation of iron-dependent PUFA causes ferroptosis. ACSL4 promotes PUFAs converted to PUFA-CoAs. PUFA-CoAs are esterified to L-PUFAs, which are oxidized by lipoxygenases leading to the generation of lipid peroxidation. System Xc- cystine/glutamate antiporter system, GSH glutathione, GPX4 glutathione peroxidase 4, Nrf2 nuclear factor erythroid 2-related factor 2, NCOA4 nuclear receptor coactivator 4, STEAP3 six transmembrane epithelial antigen of the prostate-3, TF transferrin, TFR transferrin receptor, PUFAs polyunsaturated fatty acids, ACSL4 Acyl-CoA synthetase long-chain family member 4, LOXs lipoxygenases.

Other antioxidants such as antioxidants (vitamin C, vitamin E, etc.), iron complexing agents (deferoxamine), iron inhibitors (lipoxstatin-1, ter-1) can also hinder ferroptosis. Antioxidant vitamin E can react with peroxyl free radicals to prevent the formation of lipid hydroperoxides and reduce damage. There is one experiment in which α-tocopherol, the strongest antioxidant form of vitamin E, was administered to mice with cardiac I/R injury, and the results showed that α-tocopherol significantly reduced infarct size and improved cardiac-related parameters such as ejection fraction, cardiac output [189]. This illustrates that α-tocopherol can inhibit oxidative and inflammatory responses and alleviate reperfusion injury. A previous study showed that deferoxamine as a chelating agent can reduce myocardial I/R injury [190]. In a recent study, there was no significant change in myocardial injury in ischemic-treated rats in the presence of deferoxamine; in the I/R group, ferroptosis and myocardial injury were significantly reduced in the presence of deferoxamine [191] (Fig. 5).

### Interactions between ferroptosis and other forms of cell death in myocardial I/R injury

In the complex landscape of cellular biology, ferroptosis, a distinct form of regulated cell death characterized by iron-dependent lipid peroxidation, exhibits intricate crosstalk with other important cellular processes such as apoptosis, necroptosis, and autophagy.

Ferroptosis can induce ERS-triggered apoptosis. The small-molecule inhibitors of system Xc- induce ERS, as indicated by the transcriptional upregulation of genes associated with the ERS response [192]. ERS is one of the major ways to induce apoptosis. The ERS caused by ferroptosis may lead to the occurrence of apoptosis. This mechanism of action has been verified in diabetic myocardial ischemia/reperfusion injury [193].

Ferroptosis and necroptosis are alternative. Ferroptosis is linked to necroptosis through the antagonistic relationship between ACSL4 and MLKL in acute kidney failure. After MLKL knockout, the expression of ACSL4 increases, and there is a strong correlation between them [194]. The deficiency of ACSL4 makes cells more susceptible to necroptosis and is associated with an elevation in MLKL activation [194]. However, this mechanism of action has not been demonstrated in myocardial I/R injury. Nevertheless, it still holds some suggestive significance. Heat shock protein 90, an evolutionary conserved and ubiquitously expressed molecular chaperone, serves as a common regulatory node connecting necroptosis and ferroptosis. Knockdown of heat shock protein 90 in HT-22 cells inhibits necroptosis induced by TNF-α/SM-164/z-VAD.fmk and TNF-α/cycloheximide/z-VAD.fmk, as well as ferroptosis induced by glutamate and erastin [195]. The role of heat shock protein 90 in cardiac inflammation and apoptosis has been investigated in the context of myocardial I/R injury [196]. However, its relationships with ferroptosis and pyroptosis remain unexplored.

The relationship between ferroptosis and autophagy is also very close except for apoptosis and necroptosis. Ferroptosis could be induced by the activation of autophagy in myocardial I/R injury. A study showed that autophagy-dependent ferroptosis contributes to myocardial I/R injury by overproduction of lipid signaling [197]. Conversely, inhibition of ferroptosis could be regulated by ubiquitin-specific protease 19/Beclin1-induced autophagy [132]. One of the interrelated mechanisms of ferroptosis and autophagy is the liberation of iron reservoir within ferritin proteins through ferritinophagy. NCOA4 is a crucial component of the intracellular iron homeostasis mechanism. When the cell is iron-deficient, NCOA4 can selectively recognize the ferritin heavy chain 1 in ferritin, and NCOA4 combines with the ferritin heavy chain 1 to form a complex that mediates ferritinophagy and releases ions [165]. The release of large amounts of iron by ferritin after autophagy can induce ferroptosis, causing oxidative cellular injury [198]. NCOA4-mediated ferritinophagy regulates intracellular iron levels. The overexpression of NCOA4 augments ferritin degradation and promotes ferroptosis [199]. One study showed that erastin-induced ferroptosis was promoted by increased intracellular iron content and ROS following NCOA4-mediated autophagy of ferritin [200]. This view has also been proved during myocardial I/R injury in diabetes [201].

## The key molecules mediating the interaction between different forms of cell death in myocardial I/R injury

Cell death is a complex process with significant crosstalk between different modalities. Exploring therapies that concurrently inhibit multiple cell death forms holds great promise in myocardial I/R injury. For instance, caspase-8 coordinates necroptosis and apoptosis [77], while Beclin1 and Bcl-2 interaction influences autophagy and apoptosis [116]. Caspase-3 is involved in both apoptosis and pyroptosis [143], and PANoptosis links pyroptosis, apoptosis, and necroptosis [159]. Moreover, autophagy and ferroptosis are connected in myocardial I/R injury [165]. Therefore, we further explored the impacts of several molecular targets on multiple forms of cell death in myocardial I/R injury.

Drp1 plays a crucial role in mitochondrial fission. In the cytosol, Drp1 is recruited to mitochondria through outer membrane proteins to regulate mitochondrial dynamics via Drp1 oligomerization [202]. Studies have demonstrated that phosphorylation of Drp1 at Ser616 can promote the recruitment of Drp1 to mitochondria, while AMPK agonists can prevent this process [203]. After mitochondrial division, the damaged portions are engulfed by autophagosomes. Drp1 controls autophagic flux, particularly at the level of autophagosome formation. Inhibition of Drp1 downregulates global autophagy rather than mitophagy [204]. In addition, increased phosphorylation of Drp1 at Ser616 enhance its recruitment to mitochondria following myocardial I/R injury, damaging mitochondrial respiratory function and exacerbating mitochondrial apoptosis [205]. As mentioned earlier, inhibiting Drp1 can protect myocardial cells following myocardial I/R injury by suppressing necroptosis [65]. As can be seen, these findings highlight the importance of Drp1 as a key player in multiple death pathways and emphasize its status as an important and extensively studied research target.

As autophagy promoter, AMPK activation has been proven to decrease ROS generation and prevent apoptosis and necrosis during reperfusion [206, 207]. Notably, activated AMPK can also exert a cardioprotective effect against pyroptosis. A study demonstrated that oxytocin can enhance cardiac protection and against myocardial I/R injury by suppressing pyroptosis through the AMPK signaling pathway [208]. These protective effects are partially attributed to the activation of AMPK signaling pathway. Activation of the AMPK signaling pathway can protect against ferroptosis-mediated myocardial I/R injury [209].

Studies have shown that Beclin1 was a closely related protein between autophagy and apoptosis [210]. Beclin1 interacts with Bcl-2 leads to inhibition of autophagy by forming Beclin1-Bcl-2 complex. However, rapid Bcl-2 phosphorylation disrupt the Beclin1-Bcl-2 complex and activate autophagy [211]. Besides autophagy, Beclin1 can inhibit pyroptosis by suppressing caspase-4, thereby preventing microvascular injury caused by reperfusion [212]. Moreover, a study demonstrated that the system Xc^-^ inhibitor erastin promotes the binding of Beclin1 to SLC7A11, an early step in initiating ferroptosis [213]. In other words, Beclin1 actively controls ferroptosis by directly blocking system Xc^-^ activity. Additionally, phosphorylation of MLKL is a critical step in the process of necroptosis. Beclin1 binds to MLKL in the necroptosis process, preventing the plasma membrane rupture and subsequent necroptosis [214]. However, there are no relevant reports regarding the myocardial I/R injury model.

**Fig. 6:**
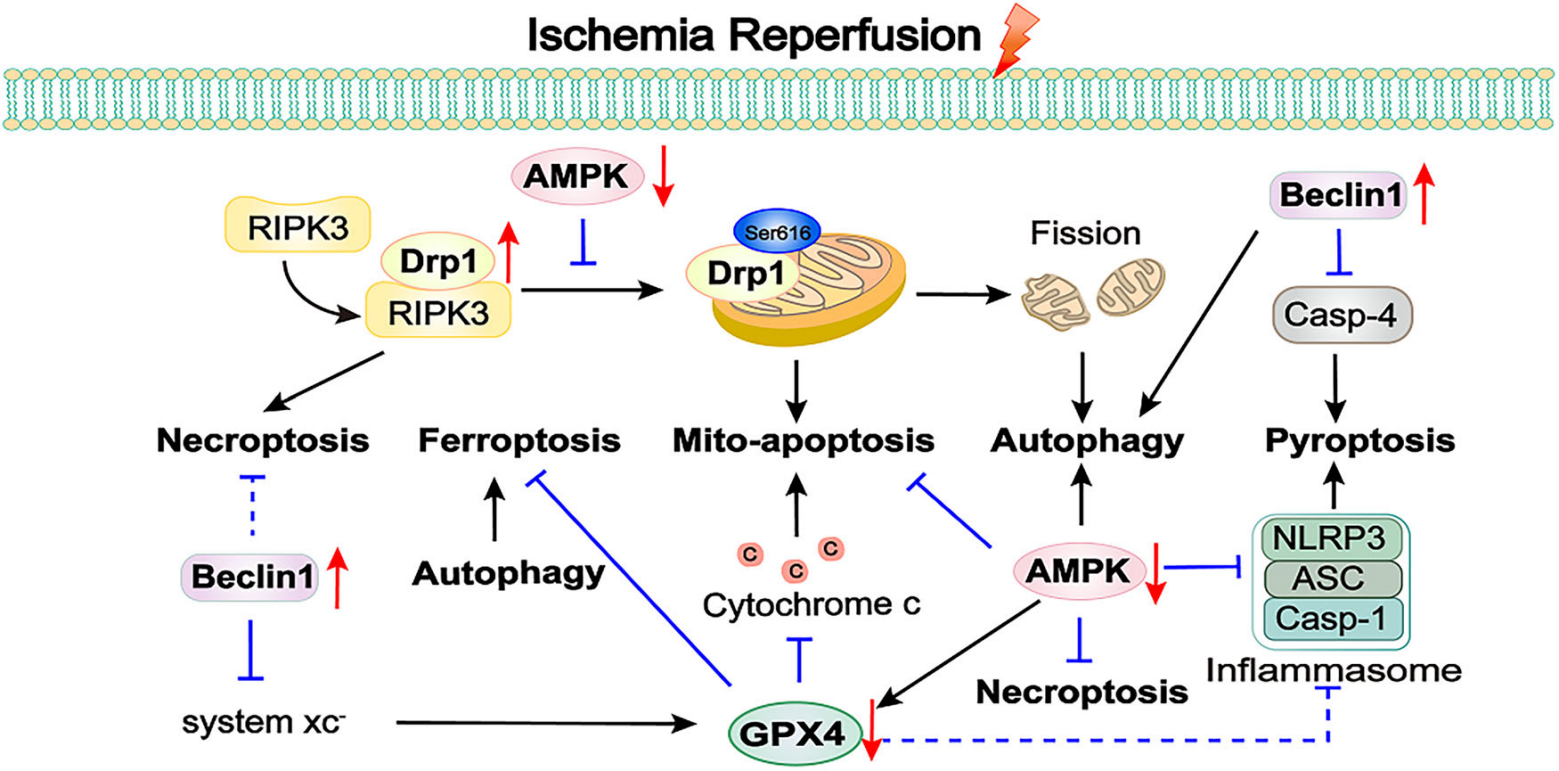
Cross-regulation of cell death pathways in myocardial I/R injury. Drp1 is phosphorylated to promote mitochondrial fission in myocardial I/R injury. The combination of Drp1 and RIPK3 exacerbates necroptosis. The excessive fission induces mito-apoptosis and the overactivation of autophagy. The later accelerating the progression of ferroptosis. However, as the autophagy promoter, AMPK could inhibit phosphorylation of Drp1 at Ser616 to exert the protective effects in myocardial I/R injury. Moreover, AMPK can inhibit apoptosis, necroptosis, pyroptosis and ferroptosis though increasing GPX4. GPX4 as one of the major antioxidant enzymes, plays a key role of ferroptosis. On the other hand, GPX4 blocks mitochondrial release of cytochrome c to reduce mito-apoptosis. GPX4 can inhibit pyroptosis in other disease models, instead of myocardial I/R injury. Beclin1 can inhibit pyroptosis by suppressing caspase-4 and control ferroptosis by directly blocking system Xc- activity. In other diseases models, Beclin1 binds to MLKL in the necroptosis process, preventing subsequent necroptosis. Drp1 dynamin-related protein 1, RIPK3 receptor-interacting protein kinase 3, AMPK AMP-activated protein kinase, System Xc- cystine/glutamate antiporter system, GPX4 glutathione peroxidase 4, NLRP3 NOD-like receptor family pyrin domain containing 3, ASC apoptosis-associated speck-like protein containing CARD, Casp caspase.

GPX4 protects myocardial cells against ROS damage-induced ferroptosis. Since both ferroptosis and mitochondrial-mediated apoptosis are caused by ROS-induced peroxides accumulation, there may be crosstalk between these two forms of cell death. In fact, inhibition of GPX4 leads to increasing mitochondrial ROS production and subsequent apoptosis in myocardial I/R injury [215]. Conversely, overexpression of GPX4 blocks mitochondrial release of cytochrome c, thereby inactivating caspase-3 [216]. GPX4 inhibition has been shown to induce increased expression of pyroptosis-related genes, including NLRP3, caspase-1, IL-18, and IL-1β in kidney injury [217]. However, there are currently no relevant research reports regarding GPX4 and pyroptosis in myocardial I/R injury, which may represent a new mechanism. Excessive activation of autophagy can also regulate ferroptosis. A recent review highlighted transmembrane protein 164-mediated autophagy as a mechanism for degrading ferritin and GPX4, thereby promoting ferroptosis [218]. Therefore, inhibition of autophagic ferroptosis would be a recent viewpoint in the treatment of myocardial I/R injury (Fig. 6).

## Expectation and discussion

As indicated above, an intricate web of connections exists among different forms of cell death. It is possible that different death inhibitors may have a synergistic effect in the treatment of myocardial I/R injury. When administered before and after ischemia, the combined use of ponatinib (necroptosis inhibitor) and deferoxamine (ferroptosis inhibitor) can suppress necroptosis and ferroptosis in the hearts of rats undergoing I/R treatment. This reduces the area of myocardial infarction and the release of creatine kinase, and the effect is more potent than using either drug alone [219]. The concurrent inhibition of necroptosis and apoptosis by means of Necrostatin-1 (necroptosis inhibitor) and Z-VAD.fmk (apoptosis inhibitor) augments the cardioprotective effect against myocardial I/R injury [220]. Recombinant apyrase (AZD3366) remarkably diminished the elevation of RIPK1, RIPK3, and p-MLKL following 1 h of reperfusion and effectively curbed the increase in IL-6 and GSDMD-N. And AZD3366 exhibited the capacity to mitigate the augmentation of these markers 24 h subsequent to reperfusion, with their effects being cumulative [221]. The results demonstrated that AZD3366 could suppress necroptosis and pyroptosis, while reducing the area of myocardial infarction. The latest research reveals that AZD3366 has entered phase I clinical trials and is well-tolerated among healthy participants [222]. The above experimental results indicate that cell deaths are interconnected due to unique factors and mechanisms, and therapies targeting multiple forms of regulated cell death hold significant clinical application prospects.

Furthermore, some key molecules have been regarded as the connections between different forms of cell death in myocardial I/R injury. For example, Drp1 links autophagy, apoptosis and necroptosis [65, 204, 205]. Inhibiting Drp1 can reduce the occurrence of autophagy, apoptosis, and necroptosis in myocardial I/R injury. In myocardial I/R injury, multiple forms of cell death such as apoptosis and necroptosis often coexist and interact with each other. Drp1 can participate in the regulation of these cell death processes through different signaling pathways. Polypeptide Globular Adiponectin, a crucial component of both the endocrine and immune systems, can suppress H/R-induced cardiomyocyte necroptosis and apoptosis through attenuating the formation of ROS, oxidative stress, and p38 MAPK and NF-κB signaling [223]. Polypeptide globular adiponectin can enhance insulin sensitivity and exert anti-inflammatory effects [224]. It can also regulate apoptosis and necroptosis during myocardial I/R injury. Since cardiovascular diseases often occur concurrently, the cumulative effects of its actions may play a more extensive protective role in patients with complex diseases. At present, it is still necessary to explore more molecules that can connect multiple forms of cell death and establish a precise interaction network, thereby enhancing the potential for developing treatment modalities by targeting key molecules.

Whether it is the combination of multiple cell death inhibitors or the targeting of molecules involved in various cell death pathways, both approaches can maximize the efficacy of therapeutic strategies, thereby effectively reducing myocardial injury and ultimately improving patient prognosis. This approach holds great promise in revolutionizing the current therapeutic landscape for myocardial-related disorders. However, there may be significant differences in the cell death regulatory mechanisms among different patients with myocardial I/R injury, which makes the precise treatment plan based on the synergy of cell death pathways highly individualized and increases the complexity of treatment.
